# Quantitative Analysis Model for the Powder Content of *Zanthoxylum bungeanum* Based on IncepSpect-CBAM

**DOI:** 10.3390/foods15010169

**Published:** 2026-01-04

**Authors:** Yue Wang, Pingzeng Liu, Sicheng Liang, Yan Zhang, Ke Zhu, Qun Yu

**Affiliations:** 1 Key Laboratory of Huang-Huai-Hai Smart Agricultural Technology, Ministry of Agriculture and Rural Affairs, Taian 271018, China; 15263068537@163.com (Y.W.); liangsichengsdau@163.com (S.L.); zhangyandxy@sdau.edu.cn (Y.Z.); kezhu@sdau.edu.cn (K.Z.); yuqun@sdau.edu.cn (Q.Y.); 2 Agricultural Big-Data Research Center, Shandong Agricultural University, Taian 271018, China; 3 School of Information Science and Engineering, Shandong Agricultural University, Taian 271018, China

**Keywords:** *Zanthoxylum* powder content detection, near-infrared spectroscopy (NIRS), convolutional block attention module (CBAM), residual network

## Abstract

The adulteration of *Zanthoxylum bungeanum* powder presents a complex challenge, as current near-infrared spectroscopy (NIRS) models are typically designed for specific adulterants and require extensive preprocessing, limiting their practical utility. To overcome these limitations, this study proposes IncepSpect-CBAM, an end-to-end one-dimensional convolutional neural network that integrates multi-scale Inception modules, a Convolutional Block Attention Module (CBAM), and residual connections. The model directly learns features from raw spectra while maintaining robustness across multiple adulteration scenarios, focusing specifically on quantifying *Zanthoxylum bungeanum* powder content. When evaluated on a dataset containing four common adulterants (corn flour, wheat bran powder, rice bran powder, and *Zanthoxylum bungeanum* stem powder), the model achieved a Root Mean Square Error of Prediction (RMSEP) of 0.058 and a coefficient of determination for prediction ($R_{P}^{2}$) of 0.980, demonstrating superior performance over traditional methods including Partial Least Squares Regression (PLSR) and Support Vector Regression (SVR), as well as deep learning benchmarks such as 1D-CNN and DeepSpectra. The results establish that the proposed model enables high-precision quantitative analysis of *Zanthoxylum bungeanum* powder content across diverse adulteration types, providing a robust technical framework for rapid, non-destructive quality assessment of powdered food products using near-infrared spectroscopy.

## 1. Introduction

*Zanthoxylum bungeanum* possesses both medicinal and culinary value. It is not only a traditional Chinese medicine [1], but also a core pungent seasoning in Sichuan cuisine [2]. Due to the growing demand and price of its processed form—*Zanthoxylum bungeanum* powder—unscrupulous vendors often adulterate it with low-cost impurities such as wheat bran powder and corn flour for profit [3,4,5], which harms consumer interests and disrupts market order. The adulteration of such powdered spices is difficult to detect visually [6], and traditional identification methods such as sensory evaluation or physicochemical analysis face limitations, including high instrument costs, subjectivity, and complex sample preparation, which makes efficient detection challenging [5]. Near-infrared spectroscopy (NIRS), by contrast, offers a promising alternative with advantages of being non-destructive, rapid, and highly sensitive [7].

Owing to its rapidity and non-destructive nature, NIRS is widely used in food adulteration detection [8,9] and has demonstrated considerable efficacy in the quantitative analysis of adulterants across diverse food matrices [10,11,12]. This is evidenced by several key applications. For example, Partial Least Squares Regression (PLSR) has been employed to quantify kernel adulteration in almond powder, achieving a high correlation (Rval > 0.96) with a low prediction error (SEP ≈ 3.98%) [13]. In quinoa flour, the optimization of PLSR using variable selection techniques yielded a highly accurate model ($R_{P}^{2}$ = 0.98, RMSEP = 1.60%) [14]. Similarly, PLSR models have been successfully applied to predict concentrations of Sudan dyes and Congo red in red chili powder [15]. Beyond PLSR, alternative algorithms like Support Vector Regression (SVR) have also shown promise, as demonstrated by Wang et al. in detecting camellia oil adulteration, where NIRS combined with SVR produced effective models for identifying corn oil ($R_{P}^{2}$ = 0.9988, RMSEP = 0.0095) and soybean oil ($R_{P}^{2}$ = 0.9984, RMSEP = 0.0117) adulterants [16]. In research on *Zanthoxylum bungeanum* powder, Wu et al. utilized PLSR to determine powder content in samples adulterated with wheat bran, rice bran, or corn flour, yielding a test set R^2^ of 0.971 [4]. These collective findings affirm the strong potential of NIRS coupled with chemometrics for quantitative adulteration analysis.

Despite considerable progress in NIRS-based food adulteration analysis, predominant methodologies remain heavily reliant on classical machine learning algorithms, particularly PLSR. A key constraint lies in PLSR’s dependence on dataset-specific preprocessing and manual feature extraction, which necessitates substantial expert intervention and yields analytical workflows that are often cumbersome and difficult to generalize [17,18,19]. Compounding this issue, the majority of existing models are calibrated for specific adulterants [20,21,22], rendering them ineffective when confronted with unexpected adulteration types. This specificity proves inadequate in practical scenarios where adulterants are diverse and unpredictable, thereby severely limiting the utility of these methods.

As a major subfield of machine learning, deep learning leverages multi-layer artificial neural networks to iteratively learn high-level representations of data, aiming primarily at prediction or classification tasks [23,24]. Recently, the integration of spectroscopy and deep learning has begun to show promising results in food adulteration detection. For example, in predicting adulteration levels in Chinese liquor, Hu et al. proposed a novel fusion network, GLSNet, which achieved an $R_{P}^{2}$ of 0.9569 ± 0.0145—outperforming traditional PLSR and improving inference efficiency by 3.55 times [25]. Moreover, Convolutional Neural Networks (CNN) have shown particular promise. Unlike manual feature extraction methods, CNNs can automatically learn deep features through convolution and pooling operations, offering improved effectiveness and robustness in regression tasks [26,27]. For instance, Zhang et al. developed the DeepSpectra model based on the Inception architecture of GoogLeNet and applied it to one-dimensional spectral analysis of small Vis-NIRS datasets involving corn, tablets, and wheat. The results showed that this model could match or even surpass the performance of PLS without any spectral preprocessing [28]. Similarly, Chakravartula et al. used FT-NIR spectroscopy combined with CNNs to quantify adulteration in commercial “espresso” coffee mixed with chicory, barley, or corn. Their model outperformed PLS and interval PLS (iPLS), achieving RMSEP values between 0.76% and 0.82% and BIASP between −0.01% and −0.10% [29].

Nevertheless, traditional CNNs still have certain limitations. Their reliance on fixed-size convolution kernels restricts the receptive field, possibly leading to incomplete extraction of global features [30]. Additionally, as the network depth increases, overfitting becomes more likely—especially on small datasets [31]. To mitigate these issues, several technical enhancements have been proposed. Attention mechanisms in deep learning emulate human visual cognition by enabling the network to focus selectively on important features in the input data [32]. Among them, the Convolutional Block Attention Module (CBAM), which integrates channel and spatial attention, dynamically adjusts the saliency of feature maps and shows potential in addressing the limitations of fixed convolution kernels and improving generalization [33]. Residual networks (ResNet) introduce direct mapping between different layers of the network, which helps deepen the architecture while maintaining performance stability and mitigating overfitting.

Based on the above research context and existing limitations, this study aims to construct a more robust quantitative analysis architecture for *Zanthoxylum bungeanum* powder adulterated with common substances such as corn flour, rice bran powder, wheat bran powder, and *Zanthoxylum bungeanum* stem powder. The proposed method seeks to minimize the need for dataset-specific preprocessing and to maintain high accuracy and generalization even in small-sample scenarios. Furthermore, this model breaks free from adulterant-type constraints, aligning more closely with practical detection needs. Accordingly, we propose the IncepSpect-CBAM model and apply it to the detection of adulteration in *Zanthoxylum bungeanum* powder. Compared with existing research, this study offers three main contributions:

(1) A one-dimensional convolutional neural network incorporating the Inception module, CBAM attention mechanism, and ResNet architecture is designed and implemented. This model eliminates the need for complex preprocessing, adapts well to small datasets, and achieves a balance between high performance and implementation simplicity.
(2) While most traditional studies on food adulteration detection focus on the content of the adulterant, this study shifts attention to detecting the content of *Zanthoxylum bungeanum* powder itself, aiming to build a generalized detection model unaffected by adulterant types. This provides a new perspective and approach for food adulteration detection.
(3) This study evaluates the proposed IncepSpect-CBAM model against several benchmark models including the 1D-CNN and DeepSpectra deep learning architectures as well as traditional PLSR and SVR methods, convincingly demonstrating its performance advantages.

## 2. Materials and Methods

### 2.1. Sample Preparation

*Zanthoxylum bungeanum* samples were collected from major production regions, including four cultivars of *Zanthoxylum bungeanum* Maxim.: Dahongpao from Hancheng (Shaanxi), Hanyuan (Sichuan), Tianshui (Gansu), and Laiwu (Shandong). These samples cover diverse geographical origins and production backgrounds, providing representative data for subsequent analysis. Four common adulterants—corn, wheat bran, rice bran, and *Zanthoxylum bungeanum* stems—were selected as adulteration substances. These materials are typical low-cost and highly concealable components frequently used in real-world adulteration of *Zanthoxylum bungeanum* powder [4,5,34], and collectively represent mainstream adulteration practices.

All samples were pulverized using a high-speed multifunctional grinder (Zhongxing Weiye, Beijing, China) and sieved through a No. 3 mesh screen (50 mesh, 0.355 mm aperture) to obtain uniform powders of pure *Zanthoxylum bungeanum*, corn, wheat bran, rice bran, and *Zanthoxylum bungeanum* stems [35], ensuring consistency and representativeness. A total of 21 predetermined adulteration levels were designed, covering a gradient from 0% to 100% at 5% intervals. For each adulterant type, samples were prepared according to these specific concentration levels, with the preparation order randomized to minimize potential systematic errors. Each sample was weighed using a high-precision analytical balance (Jiming, Shanghai, China; accuracy: 0.001 g) and homogenized using a pulse vortex mixer (BKMAM, Hunan, China) to ensure even distribution. Each prepared sample had a total mass of 2 g, yielding a total of 420 adulterated samples. All samples were sealed and stored in a desiccator after being labeled to prevent moisture interference and ensure quality.

### 2.2. Spectral Data Acquisition

Near-infrared spectral data were acquired using the NIR25S (Fuxiang, Shanghai, China) spectrometer within the 900–2500 nm range, with a sampling interval of 6.25 nm. The instrument’s HL-100 halogen light source provides continuous output from 360 to 2500 nm and is connected to the sample measurement holder via the FIB-Y-600-NIR fiber optic cable. Spectral acquisition was controlled by Morpho5 software. To effectively mitigate potential interference, strict measurement protocols were implemented: (1) The R7 holder with the fixed fiber probe was placed inside a black dark box, and all measurements were conducted in a darkroom environment at room temperature to eliminate ambient light effects; (2) a constant vertical distance of 25 mm was maintained between the fiber probe and the sample [36] to ensure consistent detection conditions; (3) cylindrical glass vessels of uniform specifications to contain powder samples, minimizing spectral errors caused by variations in sample packing thickness and compaction.

Before spectral acquisition, the light source was preheated for 30 min to ensure stable output. Prior to formal acquisition, baseline calibration was performed using the STD-WS standard reference white plate. To overcome the signal-to-noise ratio challenge posed by the weak reflected signal from the dark *Zanthoxylum bungeanum* powder sample, key acquisition parameters were optimized: the light source power was set to the maximum value permitted by the instrument, and an integration time was determined that brought the white plate signal close to saturation (approximately 60,000 counts) without overflowing. This configuration maximized initial signal intensity for low-reflectance samples while avoiding detector saturation, establishing an optimal foundation for signal-to-noise ratio. Each sample spectrum was averaged from three scans to further suppress random noise. Spectral reflectance and absorbance were calculated using the following formulas:

(1) $$R_{\lambda }=\frac{S_{\lambda }-D_{\lambda }}{W_{\lambda }-D_{\lambda }}$$

(2) $$A_{\lambda }=\log\left(\frac{1}{R_{\lambda }}\right)$$

The original spectrum contained 252 wavelength variables. To enhance the signal-to-noise ratio, segments with pronounced noise at both ends of the spectrum were excluded. Ultimately, the 1000–2400 nm spectral range was retained for subsequent modeling analysis [37], yielding a total of 420 valid spectra.

### 2.3. Sample Set Partitioning

In this study, the sample set partitioning based on joint X–Y distances (SPXY) algorithm was employed to divide the dataset into a calibration set and a prediction set, ensuring that the reference values (i.e., *Zanthoxylum bungeanum* powder content) in the prediction set fall within the range of the calibration set [38]. The calibration set was used for model training, while the prediction set served for performance evaluation. Both subsets were normalized to prevent convergence issues in the neural network caused by anomalous samples [39].

For each adulterant type, samples were independently split into calibration and prediction sets at an 8:2 ratio. The calibration set of the multi-adulterant model was built by combining the calibration subsets of all four adulteration types, and the corresponding prediction set was formed by merging the respective prediction subsets [40]. Detailed partitioning results are shown in Table 1. Both the single-adulterant models and the multi-adulterant model adopted the same dataset partitioning strategy to guarantee a fair and consistent comparison of model performance.

### 2.4. IncepSpect-CBAM Model Architecture

**Figure 1:**
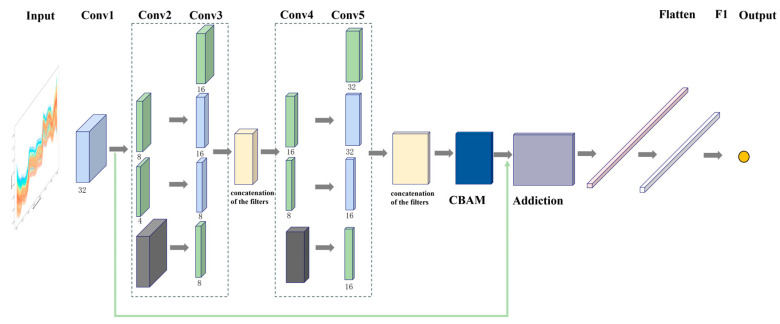
Architecture of the IncepSpect-CBAM model: The architecture comprises five convolutional layers (labeled Conv1 to Conv5), one CBAM layer, one residual connection layer, one flatten layer, one fully connected layer (F1), and one output layer. Within the convolutional layers, blue blocks represent standard convolutions, green blocks denote 1 × 1 convolutions, and gray rectangles indicate max pooling operations. Each rectangular block corresponds to a feature map. The 1 × 1 convolutions in the green blocks are used to reduce the number of channels, thereby decreasing the computational complexity of the model.

The architecture of the proposed IncepSpect-CBAM model is illustrated in Figure 1. Building upon the Inception architecture [41,42] and DeepSpectra model [28], this design incorporates specific optimizations for spectral analysis. The multi-scale Inception module simultaneously captures both local molecular vibrations and global spectral trends across different wavelength regions, while the CBAM enhances diagnostically relevant wavelengths through adaptive feature optimization. Residual connections ensure stable training of the deep network and mitigate overfitting risks in small-sample datasets. This integrated design provides a systematic solution that reduces dependence on manual preprocessing while enabling accurate prediction of *Zanthoxylum bungeanum* powder content directly from raw spectral inputs. The model accepts one-dimensional raw spectral data and outputs the target concentration through a core architecture comprising five convolutional layers, one CBAM, residual connections, and fully connected layers.

The initial convolutional layer Conv1 employs 32 convolution kernels of identical size, with a kernel size of 7 and a stride of 3. This configuration is designed to capture the local and continuous features in spectral data using a relatively large kernel, while simultaneously reducing the spatial dimension of the feature maps through strided convolution, thereby lowering computational complexity. After this layer, the one-dimensional spectral input is transformed into 32 feature maps of reduced dimensionality.

In the multi-branch module design, Inception modules are embedded between Conv2 and Conv3, as well as between Conv4 and Conv5, forming two complete Inception blocks connected in series [28,41]. Each Inception block contains four parallel convolutional branches with different kernel sizes, along with max pooling and 1 × 1 convolutions. This design simultaneously increases both the depth and width of the spectral analysis model—enhancing its ability to extract abstract and complex features, thus improving the model’s fitting performance. In the architecture, green modules represent 1 × 1 convolutions, gray modules denote max pooling layers, and blue modules indicate standard convolutions. The combination of 1 × 1 convolutions and pooling operations helps reduce both the number of parameters and the length of the feature maps. Notably, Conv3 and Conv5 adopt three different kernel sizes, allowing the model to extract spectral features at multiple scales simultaneously, which enhances the adaptability of the CNN to spectral variability.

**Figure 2:**
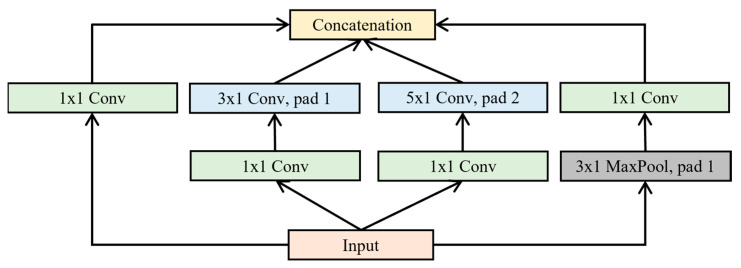
Inception structure.

Specifically, each Inception block extracts multi-scale information through four separate branches, which are finally merged along the channel dimension, as illustrated in Figure 2. The first three branches consist of convolutional layers with 1 × 1, 3 × 1, and 5 × 1 kernels, respectively, capturing features at different spatial scales. The fourth branch performs max pooling, followed by a 1 × 1 convolution to adjust the number of channels. All four branches adopt appropriate padding strategies to ensure that the input and output share the same height and width dimensions. The outputs from the four branches are concatenated along the channel axis to form the final output of the Inception block. The main tunable hyperparameter of each Inception block is the number of output channels for each branch.

**Figure 3:**
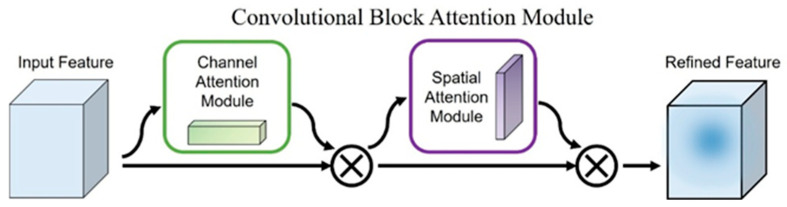
Structure of the CBAM.

To enhance the model’s focus on critical spectral bands, a CBAM is embedded after the second Inception block in the IncepSpect-CBAM architecture. The structure of CBAM is illustrated in Figure 3. CBAM consists of a channel attention module and a spatial attention module, which together enable the model to concentrate on the most informative features. As a lightweight and generic module, CBAM can be seamlessly integrated into any convolutional architecture and trained end-to-end.

In the channel attention module, average pooling and max pooling operations are performed separately on the input feature map to extract spatial information. The resulting descriptors are then passed through a multi-layer perceptron (MLP) in the hidden layer to perform dimensionality reduction or expansion. The outputs of the two pooling operations are summed element-wise and processed by an activation function to generate the final channel attention map. The computation is defined in Equation (3):

(3) $$M_{c}(F)=\delta \left(MLP\left[\left(F_{\mathrm{avg}}^{c}\right)+\left(F_{\max}^{c}\right)\right]\right)$$

Spatial attention module serves as a complement to the channel attention module. It performs average pooling and max pooling operations along the channel dimension of the input feature map, resulting in two single-channel feature maps. These two maps are then concatenated, followed by a convolution operation, and finally passed through an activation function to generate the spatial attention map. The computation is defined in Equation (4):

(4) $$M_{s}(F)=\delta (f^{i\times i}[(F_{\mathrm{avg}}^{s});(F_{\max}^{s})])$$

Here, F denotes the input feature map, and δ represents the Sigmoid activation function.

In addition, a residual connection is introduced into the model to alleviate the problems of gradient vanishing and gradient explosion in deep neural networks. In this architecture, the residual block performs an identity mapping between shallow and deep network layers via skip connections (as illustrated by the green line at the bottom of Figure 1). Specifically, the output from one or more preceding layers is added to the output of the current layer, and the sum is then passed through an activation function. This design guarantees that the worst-case performance of residual learning is no worse than the output of the previous layer, effectively improving both the training efficiency and the feature representation capability of the model [40].

At the end of the network, a flatten layer and a fully connected (FC) layer are included, along with a Dropout strategy for performance optimization. The flatten layer concatenates and flattens the output feature maps from the previous layer into a one-dimensional vector, which is then fed into the FC layer. To suppress overfitting and enhance computational accuracy, Dropout is applied with a dropout rate of 20%, temporarily deactivating a subset of neurons during each training iteration, thus reducing the number of parameters being trained. The FC layer contains fewer neurons than the flatten layer and connects to the output layer through a fully connected structure. Since the model predicts a single target value, the output layer consists of one node, whose value corresponds to the predicted variable.

Before training the CNN, a loss function must be defined to quantify the error between the predicted and true values. When the error drops below a predefined threshold, the model is considered to have achieved satisfactory performance, and training is terminated. In this study, the Mean Squared Error (MSE) is employed as the loss function. To mitigate overfitting, L2 regularization is also introduced to constrain the model’s weight parameters. The complete loss function is defined in Equation (5):

(5) $$Loss=\frac{1}{N}\sum_{n=1}^{N}\left[{\left(y_{n}-{\hat{y}}_{n}\right)}^{2}\right]+\lambda \|w{\|}^{2}$$

Here, $y_{n}$ and ${\hat{y}}_{n}$ represent the true value and predicted value, respectively; N denotes the number of training samples; w is the weight matrix, and λ is the regularization coefficient.

To introduce nonlinearity into the network, an activation function is applied after each convolutional and fully connected layer. In this study, the Mish activation function is adopted. The computation of the Mish function is defined in Equation (6):

(6) $$f(x)=x*\tanh\left(\ln(1+e^{x})\right)$$

To alleviate gradient vanishing and improve the generalization ability of the network, Batch Normalization (BN) layers were introduced after convolutional layers, the flatten layer, and the fully connected layer to accelerate network convergence. The batch size and initial learning rate were optimized through a combination of preliminary trial-and-error and grid search. The batch size was set to 32, and the initial learning rate to 0.001. The model was trained using the Backpropagation (BP) algorithm combined with the AdamW optimizer, aiming to minimize the loss function and find its local optimum.

### 2.5. Quantitative Prediction Models for Comparison

#### 2.5.1. DeepSpectra Model

The proposed IncepSpect-CBAM model was compared against the DeepSpectra model. It serves as a well-established end-to-end deep learning baseline in spectral analysis, which incorporates fundamental Inception modules to capture multi-scale features. To ensure a fair and rigorous comparison, we conducted a systematic optimization of the DeepSpectra architecture. A comprehensive grid search with 5-fold cross-validation was performed, exploring key architectural and training hyperparameters to identify the optimal configuration for our dataset. The final, optimized architecture used for all comparative evaluations is detailed in Table 2.

#### 2.5.2. 1 D-CNN Model

A 1D-CNN was implemented as an additional baseline to enable a broader comparison of architectural complexity against simpler deep learning models. This model represents a fundamental convolutional network with a straightforward sequential structure, providing a reference for the performance gains achieved by more sophisticated architectural designs. This network comprises three sequential 1D convolutional layers, an adaptive max pooling layer, and three fully connected layers. To ensure its performance was fully realized and the comparison was equitable, the model’s architecture and hyperparameters were systematically optimized via an extensive grid search with 5-fold cross-validation. The optimal configuration determined through this process is comprehensively detailed in Table 3.

#### 2.5.3. Traditional Quantitative Modeling Methods

To ensure a rigorous and fair comparison with the proposed deep learning model, we systematically optimized the traditional methods—PLSR and SVR—to ensure their best possible performance. These models represent classical approaches that typically require spectral preprocessing and feature selection to achieve optimal performance. These models were evaluated under multiple spectral preprocessing and feature selection strategies, including Multiplicative Scatter Correction (MSC), Standard Normal Variate (SNV), Competitive Adaptive Reweighted Sampling (CARS), and Successive Projection Algorithm (SPA).

For PLSR, the optimal number of latent variables (LVs) was determined through 5-fold cross-validation, searching within a range of 5 to 20. For SVR with a Radial Basis Function (RBF) kernel, a two-step grid search was conducted to identify the best combination of the penalty parameter (C) and the kernel coefficient (gamma). The key hyperparameters for both models, corresponding to their optimal performance under each data processing strategy, are summarized in Table 4.

### 2.6. Model Performance Evaluation Metrics

The predictive performance of the models was assessed using the following evaluation metrics including Coefficient of Determination (R^2^), Root Mean Square Error (RMSE), and Residual Predictive Deviation (RPD). The closer the R^2^ value is to 1, the better the model’s fitting performance, which indicates a stronger correlation between spectral features and the target component. A smaller RMSE indicates lower prediction error and higher accuracy. RPD is used to assess the stability and predictive capability of the model, and a higher RPD implies stronger model robustness. Specifically, RPD < 2.4 means poor model reliability, 2.4 ≤ RPD < 3.0 represents acceptable prediction performance, and RPD ≥ 3.0 indicates excellent prediction ability [33].

## 3. Results and Discussion

### 3.1. Spectral Feature Analysis

**Figure 4:**
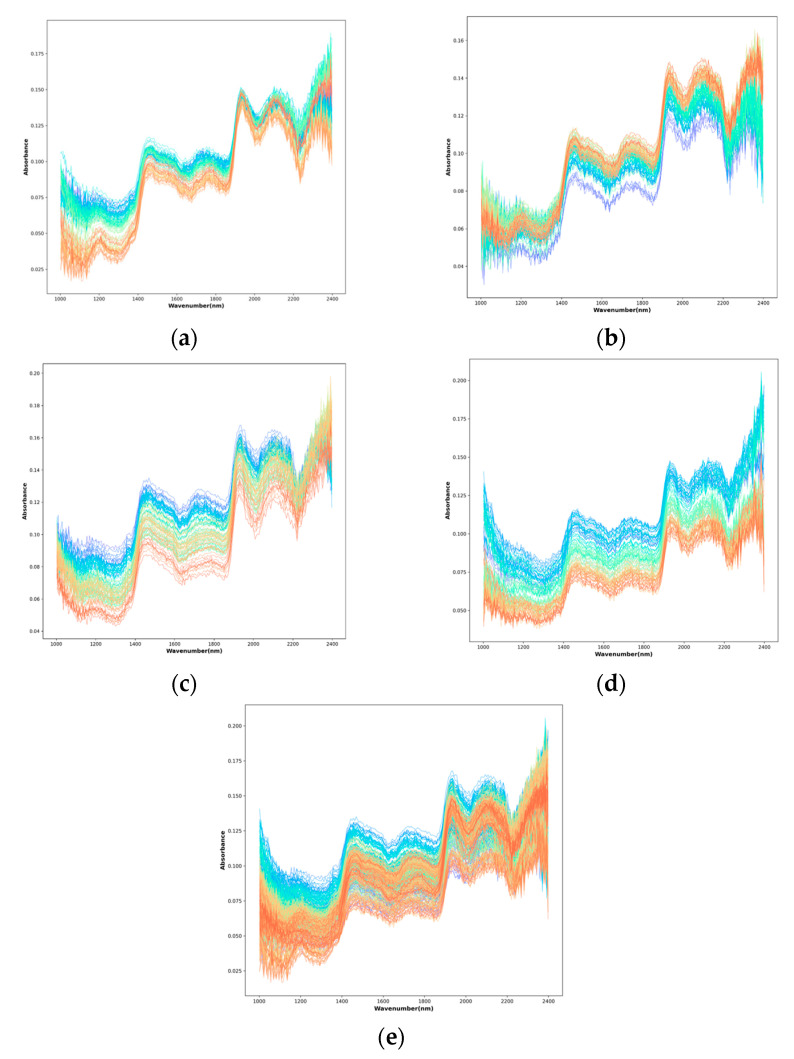
Spectral data of samples with different adulteration types, where (**a**) denotes samples adulterated with corn flour; (**b**) denotes samples adulterated with wheat bran flour; (**c**) denotes samples adulterated with rice bran flour; (**d**) denotes samples adulterated with *Zanthoxylum bungeanum* stem powder; (**e**) denotes mixed samples.

Figure 4a–e present the raw near-infrared spectra of *Zanthoxylum bungeanum* powder samples adulterated with the four individual types of adulterants, as well as the combined dataset of all samples. As shown in the figures, distinct absorption peaks appear around 1200 nm, 1420 nm, 1700 nm, 1900 nm, and 2100 nm. Specifically, the peaks at 1200 nm and 1700 nm are likely associated with the second and third overtone absorptions of C–H bonds, while the absorption features near 1420 nm and 1900 nm are likely caused by water content in the samples—which exhibits characteristic absorption in the near-infrared region [4]. Although differences in absorbance intensity are observed among samples with different adulterant types, the overall peak shapes and positions remain similar. Furthermore, due to substantial spectral overlap, it is difficult to distinguish between different adulteration types based solely on peak locations or visual spectral features. Therefore, it is necessary to employ machine learning-based quantitative analysis methods to accurately detect the *Zanthoxylum bungeanum* powder content.

### 3.2. Performance Comparison with Baseline Models

#### 3.2.1. Comparative Analysis of Deep Learning Models

**Figure 5:**
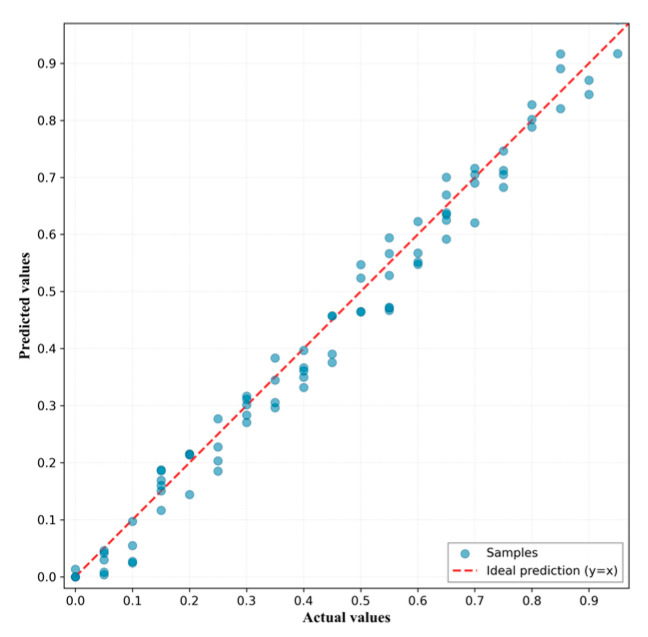
Scatter plot of predicted versus actual values for the IncepSpect-CBAM model.

The quantitative analysis results of different deep learning models on raw spectral data are presented in Table 5. The proposed IncepSpect-CBAM model achieved optimal performance on raw spectral data, with an RMSEP of 0.058 and $R_{P}^{2}$ of 0.980. The scatter plot of predicted versus actual values is shown in Figure 5.

Performance comparison revealed that IncepSpect-CBAM reduced RMSEP by 46.3% and 25.6% compared to 1D-CNN and DeepSpectra, respectively, while improving $R_{P}^{2}$ by 8.5% and 3.2%. Furthermore, IncepSpect-CBAM achieved an RPD value of 6.203, higher than both 1D-CNN (RPD = 3.189) and DeepSpectra (RPD = 4.105), demonstrating better model robustness.

As the second-best performing model, DeepSpectra achieved an RMSEP of 0.078 and an $R_{P}^{2}$ of 0.950. Compared with 1D-CNN (RMSEP = 0.108, $R_{P}^{2}$ = 0.903), DeepSpectra reduced RMSEP by 27.8% and improved $R_{P}^{2}$ by 5.2%, validating the advantage of multi-scale convolutional structures in spectral feature extraction.

The observed performance differences originate from the distinctive design characteristics of each model architecture. The 1D-CNN, constrained by its limited receptive field, exhibits deficiencies in comprehensively capturing both local spectral details and global trends. DeepSpectra employs Inception modules that enhance feature extraction capability through parallel multi-scale convolution, consistent with findings from Zhang et al. [28]. However, this model still lacks a screening mechanism for identifying key spectral regions.

The CBAM attention mechanism introduced in this study enables automatic focus on feature bands closely associated with *Zanthoxylum bungeanum* powder content through dynamic weight adjustment, thereby enhancing the capability to distinguish different components within complex spectral backgrounds. This finding aligns with conclusions from recent research [33] regarding the effectiveness of attention mechanisms in optimizing feature selection. Additionally, residual connections ensure stable optimization of the deep architecture under limited training sample conditions, thereby supporting adequate training of the complex model.

#### 3.2.2. Benchmarking Against Traditional Chemometric Methods

Table 6 presents the performance comparison of the IncepSpect-CBAM model against two traditional chemometric methods under different data processing strategies. The IncepSpect-CBAM model achieved optimal prediction results using raw spectral data ($R_{P}^{2}$ = 0.980, RMSEP = 0.058). Its predictive performance with various preprocessing and feature selection methods (RMSEP range: 0.062–0.115) did not exceed that achieved with raw data, indicating the model’s capability for end-to-end analysis through autonomous learning from raw spectra.

**Figure 6:**
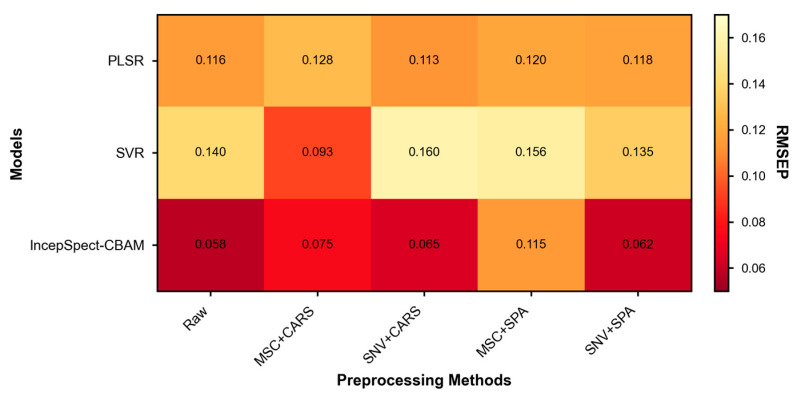
Effect of preprocessing and variable selection methods on model prediction accuracy.

The performance of traditional chemometric methods demonstrated dependence on specific preprocessing combinations, as visualized in Figure 6. PLSR achieved optimal performance under the SNV + CARS strategy ($R_{P}^{2}$ = 0.893, RMSEP = 0.113), while SVR only reached its best performance with the MSC + CARS combination ($R_{P}^{2}$ = 0.914, RMSEP = 0.093). The same preprocessing method produced distinct effects on different models, as exemplified by SNV + CARS enhancing PLSR performance while increasing SVR’s RMSEP to 0.160. These variations indicate that traditional methods require specialized optimization for different algorithms.

The RMSEP obtained by IncepSpect-CBAM using raw data was 48.7% and 37.6% lower than the optimized PLSR and SVR models, respectively. This performance difference stems from model architecture characteristics. PLSR, as a linear model, exhibits limitations in handling nonlinear spectral responses in complex powder systems. Although SVR can manage nonlinear relationships, its effectiveness depends heavily on preprocessing and feature selection strategies, consistent with findings from Vera et al. [43]. IncepSpect-CBAM achieves multi-scale feature extraction through its end-to-end deep architecture and employs the CBAM attention mechanism for dynamic weight adjustment to focus on critical spectral regions, thereby reducing reliance on manual preprocessing.

**Figure 7:**
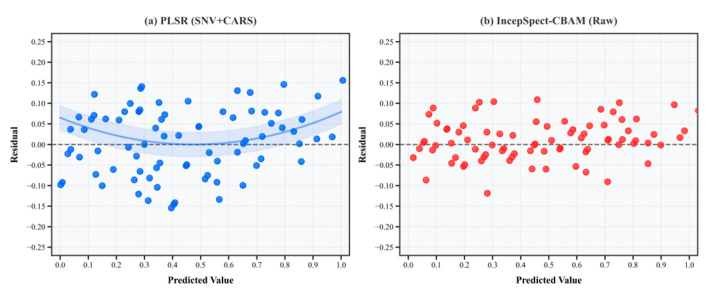
Comparison of residual distributions between PLSR and IncepSpect-CBAM models.

To visually elucidate the underlying causes of the aforementioned performance differences, we compared the residual distributions of the optimally preprocessed PLSR model and the proposed IncepSpect-CBAM model using raw spectra on the prediction set, as shown in Figure 7. The PLSR residual plot reveals a distinct systematic bias, characterized by an approximately U-shaped distribution of residuals across the concentration gradient. Specifically, the model systematically overestimates the target values in the mid-concentration range while systematically underestimating them at both high and low concentration extremes. This non-random residual structure indicates that the linear PLSR model fails to adequately capture the nonlinear relationships inherent in the data. In contrast, the residuals of the IncepSpect-CBAM model are randomly and uniformly distributed around the zero-line across the entire concentration range, showing no apparent systematic bias. This clearly demonstrates that its nonlinear architecture effectively learns and compensates for complex nonlinear effects.

Methodologically, this study establishes a meaningful comparison with the work of Wu et al. [4]. Their research achieved quantitative analysis of *Zanthoxylum* powder content in mixed adulterants using PLSR ($R_{P}^{2}$ = 0.971). While maintaining comparable accuracy, our study demonstrates the unique advantages of deep learning architecture through the development of a quantitative analysis model that operates without complex preprocessing, providing a new technical pathway for addressing the challenge of unknown adulterants in practical detection scenarios.

### 3.3. Ablation Study Analysis

To quantitatively evaluate the contribution of each architectural component in the proposed IncepSpect-CBAM model, systematic ablation studies were conducted. As detailed in Table 7, the removal of any major component led to significant and systematic performance degradation. Most notably, eliminating the CBAM attention mechanism resulted in the most pronounced reduction in performance, with $R_{P}^{2}$ decreasing from 0.980 to 0.955 and RPD declining from 6.203 to 4.721. This underscores the critical role of CBAM in adaptively directing computational resources toward the most diagnostically significant spectral regions while suppressing irrelevant variations.

Similarly, replacing the multi-scale Inception modules with standard convolutional layers caused a substantial performance decline, reducing $R_{P}^{2}$ from 0.980 to 0.962, thereby validating the necessity of simultaneous multi-scale feature extraction for capturing both local molecular vibrations and broader spectral trends in complex powder mixtures. Furthermore, although the impact was relatively modest, removing the residual connections still resulted in measurable performance deterioration, lowering $R_{P}^{2}$ from 0.980 to 0.972, which confirms their importance in maintaining training stability and gradient flow in deep networks.

Collectively, the consistent performance degradation observed across all ablation scenarios demonstrates that each component makes unique and indispensable contributions to the model’s final predictive capability.

### 3.4. Comparison Between Single-Adulterant and Multi-Adulterant IncepSpect-CBAM Models

Table 8 summarizes the performance of single-adulterant and multi-adulterant IncepSpect-CBAM models across key evaluation metrics. Among the single-adulterant models, the model trained on corn flour adulteration achieved the highest accuracy ($R_{P}^{2}$ = 0.977, RMSEP = 0.060), which can be attributed to the distinct spectral characteristics between corn flour and *Zanthoxylum bungeanum* powder. The other three single-adulterant models performed significantly worse than the multi-adulterant model, likely because their spectral differences from pure *Zanthoxylum bungeanum* powder were less pronounced, making discriminative feature extraction more challenging.

The multi-adulterant model consistently yielded lower prediction errors than any single-adulterant model, with an RMSEP of 0.058 compared to the range of 0.060–0.089 for single-adulterant models. From the perspective of RMSECV, the multi-adulterant model also demonstrated superior performance with the lowest value (0.055). This advantage is attributed to the multi-adulterant model’s training set, which contained samples with diverse adulteration types, providing richer spectral information. Consequently, the multi-adulterant model could better adapt to different adulteration scenarios, while single-adulterant models were limited in adaptability due to their narrow training scope. The multi-adulterant model achieved an RPD value of 6.203, indicating strong practical utility and reaffirming the well-established machine learning principle that a larger and more diverse training set generally leads to better model performance.

This finding aligns with conclusions from other spectral deep learning studies [40], where expanding the diversity of the calibration dataset enhanced model robustness against unseen variations. Future work will therefore focus on increasing both the volume and diversity of samples to further improve model generalization.

## 4. Conclusions

This study proposed an end-to-end deep learning architecture, IncepSpect-CBAM, for the quantitative analysis of *Zanthoxylum bungeanum* powder content. The model integrates multi-scale Inception modules, a Convolutional Block Attention Module (CBAM), and residual connections, enabling it to learn directly from raw NIR spectra without relying on complex preprocessing or manual feature selection. With raw spectral input, the model achieved superior performance ($R_{P}^{2}$ = 0.980, RMSEP = 0.058, RPD = 6.203), significantly outperforming traditional PLSR and SVR models, as well as deep learning baseline models including 1D-CNN and DeepSpectra. Ablation studies quantitatively confirmed the critical contribution of each core component. Furthermore, the model demonstrated exceptional generalization capability across multiple adulterants, outperforming models trained on single adulterant types and validating the paradigm of building a universal detector focused on the target component, independent of adulterant types.

Future work will focus on expanding the research boundaries by increasing the diversity of pure samples to include cultivars from more geographical origins, while also employing external validation sets comprising independent batches and entirely new types of adulterants. This approach will enable a systematic evaluation of the model’s generalizability and predictive performance in real-world, open-scenario applications. The architectural framework presented in this study provides a viable technical solution for rapid, non-destructive quality detection of powdered foods.

## Figures and Tables

**Table 1:** Detailed sample partitioning.

Type	Set	Number	Range
corn flour	Calibration	84	0~100%
	Prediction	21	0~95%
wheat bran powder	Calibration	84	0~100%
	Prediction	21	0~75%
rice bran powder	Calibration	84	0~100%
	Prediction	21	0~85%
*Zanthoxylum bungeanum* stem powder	Calibration	84	0~100%
	Prediction	21	10~95%
Combined (Multi-Adulterant)	Calibration	336	0~100%
	Prediction	84	0~95%

**Table 2:** Core parameters of the DeepSpectra model.

Module	Parameter Name	Parameter Value
Input	Spectral Length	213
Conv1	Kernel Size/Stride/Filters	5/3/4
Inception	Branch 1 Kernels	1, 3
	Branch 2 Kernels	1, 5
	Branch 3 Kernels	3, 3
	Output Channels	8, 8, 8
FC	FC1 Neurons	100
Output	Output Neurons	1
Regularization	Dropout Rate	0.1
Training	Learning Rate	0.001
	Batch Size	32

**Table 3:** Core parameters of the 1D-CNN baseline model.

Module	Parameter Name	Parameter Value
Input	Spectral Length	213
Conv1	Kernel Size/Stride/Filters	7/1/16
Conv2	Kernel Size/Stride/Filters	5/1/32
Conv3	Kernel Size/Stride/Filters	3/1/64
Pooling	Adaptive Max Pooling Output Size	128
FC	FC1/FC2/FC3 Neurons	8192/128/64
Output	Output Neurons	1
Regularization	Dropout Rate	0.2
	L2 Regularization Coefficient	0.001
Training	Learning Rate	0.001
	Batch Size	32

**Table 4:** Optimal hyperparameters for traditional models under different preprocessing and feature selection strategies.

Model	Processing Strategy	Optimal Hyperparameters
PLSR	Raw	LVs: 12
	MSC + CARS	LVs: 8
	SNV + CARS	LVs: 10
	MSC + SPA	LVs: 9
	SNV + SPA	LVs: 11
SVR	Raw	C: 10, gamma: 0.01
	MSC + CARS	C: 100, gamma: 0.001
	SNV + CARS	C: 10, gamma: 0.01
	MSC + SPA	C: 100, gamma: 0.01
	SNV + SPA	C: 10, gamma: 0.1

**Table 5:** Performance comparison of deep learning models on raw spectral data.

Model	$R_{C}^{2}$	RMSECV	$R_{P}^{2}$	RMSEP	RPD
1D-CNN	0.925	0.105	0.903	0.108	3.189
DeepSpectra	0.962	0.079	0.950	0.078	4.105
IncepSpect-CBAM	0.985	0.055	0.980	0.058	6.203

**Table 6:** Performance comparison of different modeling methods under various preprocessing strategies.

Model	Methods	$R_{C}^{2}$	RMSECV	$R_{P}^{2}$	RMSEP	RPD
PLSR	Raw	0.899	0.127	0.891	0.116	3.073
	MSC + CARS	0.858	0.132	0.851	0.128	2.812
	SNV + CARS	0.902	0.118	0.893	0.113	3.126
	MSC + SPA	0.870	0.135	0.860	0.120	2.907
	SNV + SPA	0.885	0.130	0.875	0.118	2.951
SVR	Raw	0.908	0.120	0.743	0.140	1.971
	MSC + CARS	0.911	0.115	0.914	0.093	3.411
	SNV + CARS	0.908	0.103	0.597	0.160	1.574
	MSC + SPA	0.737	0.149	0.632	0.156	1.648
	SNV + SPA	0.925	0.098	0.776	0.135	2.113
IncepSpect-CBAM	Raw	0.985	0.055	0.980	0.058	6.203
	MSC + CARS	0.960	0.075	0.950	0.075	4.806
	SNV + CARS	0.970	0.065	0.960	0.065	4.904
	MSC + SPA	0.940	0.110	0.930	0.115	4.509
	SNV + SPA	0.975	0.060	0.970	0.062	5.046

**Table 7:** Results of ablation experiments on the IncepSpect-CBAM model.

Model	$R_{C}^{2}$	RMSECV	$R_{P}^{2}$	RMSEP	RPD
Proposed (Full)	0.985	0.055	0.980	0.058	6.203
w/o CBAM	0.963	0.078	0.955	0.082	4.721
w/o Inception	0.970	0.070	0.962	0.075	4.987
w/o Residual	0.975	0.065	0.972	0.068	5.312

**Table 8:** Performance comparison between single-adulterant and multi-adulterant IncepSpect-CBAM models.

Type	$R_{C}^{2}$	RMSECV	$R_{P}^{2}$	RMSEP	RPD
corn flour	0.955	0.065	0.977	0.060	6.171
wheat bran powder	0.919	0.121	0.912	0.079	3.365
rice bran powder	0.923	0.084	0.915	0.072	3.436
*Zanthoxylum bungeanum* stem powder	0.872	0.099	0.917	0.089	3.464
Combined (Multi-Adulterant)	0.985	0.055	0.980	0.058	6.203

## Data Availability

The original contributions presented in the study are included in the article, further inquiries can be directed to the corresponding author.

## Abbreviations

The following abbreviations are used in this manuscript:

NIRS	Near-infrared spectroscopy
CBAM	Convolutional Block Attention Module
PLSR	Partial Least Squares Regression
SVR	Support Vector Regression
